# Remote monitoring of cardiac implantable electronic devices using smart device interface versus radiofrequency‐based interface: A systematic review

**DOI:** 10.1002/joa3.13054

**Published:** 2024-05-09

**Authors:** Vern Hsen Tan, Hui Xin See Tow, Khi Yung Fong, Yue Wang, Colin Yeo, Chi Keong Ching, Toon Wei Lim

**Affiliations:** ^1^ Department of Cardiology Changi General Hospital Singapore Singapore; ^2^ Yong Loo Lin School of Medicine National University of Singapore Singapore Singapore; ^3^ Department of Cardiology National Heart Centre Singapore Singapore Singapore; ^4^ Department of Cardiology National University Heart Centre Singapore Singapore Singapore

**Keywords:** Bluetooth, cardiac implantable electronic devices, remote monitoring, smartphone

## Abstract

**Background:**

Guidelines recommended remote monitoring (RM) in managing patients with Cardiac Implantable Electronic Devices. In recent years, smart device (phone or tablet) monitoring‐based RM (SM‐RM) was introduced. This study aims to systematically review SM‐RM versus bedside monitor RM (BM‐RM) using radiofrequency in terms of compliance, connectivity, and episode transmission time.

**Methods:**

We conducted a systematic review, searching three international databases from inception until July 2023 for studies comparing SM‐RM (intervention group) versus BM‐RM (control group).

**Results:**

Two matched studies (21 978 patients) were retrieved (SM‐RM arm: 9642 patients, BM‐RM arm: 12 336 patients). There is significantly higher compliance among SM‐RM patients compared with BM‐RM patients in both pacemaker and defibrillator patients. Manyam et al. found that more SM‐RM patients than BM‐RM patients transmitted at least once (98.1% vs. 94.3%, *p* < .001), and Tarakji et al. showed that SM‐RM patients have higher success rates of scheduled transmissions than traditional BM‐RM methods (SM‐RM: 94.6%, pacemaker manual: 56.3%, pacemaker wireless: 77.0%, defibrillator wireless: 87.1%). There were higher enrolment rates, completed scheduled and patient‐initiated transmissions, shorter episode transmission time, and higher connectivity among SM‐RM patients compared to BM‐RM patients. Younger patients (aged <75) had more patient‐initiated transmissions, and a higher proportion had ≥10 transmissions compared with older patients (aged ≥75) in both SM‐RM and BM‐RM groups.

**Conclusion:**

SM‐RM is a step in the right direction, with good compliance, connectivity, and shorter episode transmission time, empowering patients to be in control of their health. Further research on cost‐effectiveness and long‐term clinical outcomes can be carried out.

## Graphical abstract

Our systematic review concludes that smart device remote monitoring is a step in the right direction, with good compliance, connectivity, and shorter episode transmission time, empowering patients to be in control of their health. Further research on cost‐effectiveness and long‐term clinical outcomes can be carried out.

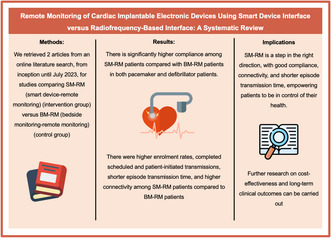

## 1 INTRODUCTION

Remote monitoring (RM) is the standard of care in managing patients with Cardiac Implantable Electronic Devices (CIED). The benefits of RM include rapid response to events (arrhythmic events, lead or device malfunction, patient's initiated event), reduced need for in‐person evaluations,^1^, ^2^, ^3^, ^4^ cost reduction, and mortality benefit.^1^, ^5^, ^6^ Guidelines advocate that all patients with CIEDs should be offered RM as part of the standard follow‐up management strategy.^7^, ^8^

In the late 1990s, inductive technology was incorporated into the field of CIEDs.^9^ This technology utilizes a wand‐based frequency platform to transfer data between the patient's device and a home transceiver, via a telephone line to a central repository. However, this system can be time‐consuming and cumbersome, lacking automatic transmission for asymptomatic events. In 2001, fully automatic RM platforms for RM were introduced, enabling regular transmissions initiated by the device if abnormal criteria are detected or if patients triggered transmissions based on their symptoms.^10^ The data were transmitted wirelessly to a transceiver located close to the patient, then to the manufacturer's central repository using either analogue landlines or a wireless data network, and retrieved by healthcare providers such as physicians or cardiac physiologists. RM offers the benefits of frequent data updates without constant patient presence, facilitating timely interventions by healthcare providers. These benefits were significant during the COVID‐19 pandemic, which has led to a significant increase in the use of RM of CIEDs.^11^, ^12^, ^13^ However, radiofrequency technology is limited by the external transmitter, which needs to be placed at a specific location (usually the patient's bedroom) to allow the device to communicate with the patient's CIED. This inconveniences patients, especially post‐pandemic, for patients who travel frequently. Other barriers to its use include the lack of enrolment of eligible patients, institutions or physicians' practice, geographical factors, socioeconomic status, or patient‐related factors such as a desire to have in‐person visits with the healthcare provider.^14^ The continuity of monitoring is paramount, as a study has shown that patients who adhere to RM and transmit data consistently are at substantially lower risk of death and hospital readmission.^14^

In recent years, smart device‐based RM (SM‐RM) was introduced. SM‐RM is a novel technology that uses Bluetooth low‐energy (BLE), a cellular or Wi‐Fi internet connection, and a specifically developed smart device (phone or tablet) application connected to the manufacturer's repository. The smart device is either provided by a manufacturer that is pre‐configured or a patient's smart device (either Android or iOS operating system). Patients can download and install the application from the App Store on their smart devices. Using BLE, the technology enables data encryption, automatic and secure communication with their CIEDs while adhering to privacy regulations like the Health Insurance Portability and Accountability Act (HIPAA).^15^ However, the impact of SM‐RM on compliance and connectivity remains unknown when compared to radiofrequency induction‐based RM.

This study aims to perform a systematic review on SM‐RM versus bedside monitor RM (BM‐RM) in terms of compliance, connectivity, and episode transmission time.

## 2 METHODS

This systematic review adhered to the Preferred Reporting Items for Systematic Reviews and Meta‐Analyses (PRISMA) guidelines.^16^ Ethics approval and patient consent were not required because this study used publicly available data, and no human subjects were involved.

### 2.1 Literature search

**FIGURE 1:**
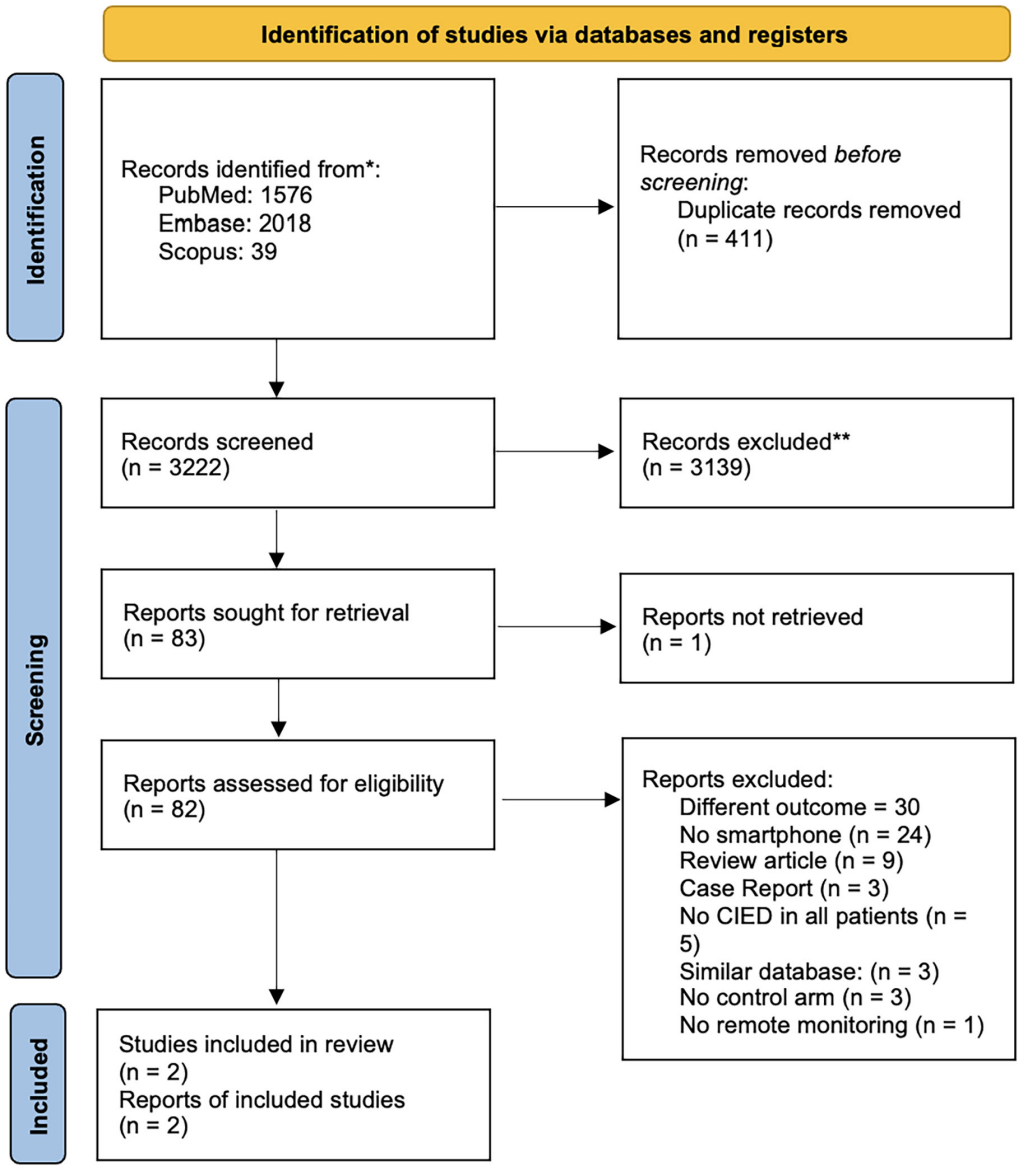
PRISMA flowchart. *Consider, if feasible to do so, reporting the number of records identified from each database or register searched (rather than the total number across all databases/registers). **If automation tools were used, indicate how many records were excluded by a human and how many were excluded by automation tools. *Source*: Page MJ, McKenzie JE, Bossuyt PM, Boutron I, Hoffmann TC, Mulrow CD, et al. The PRISMA 2020 statement: an updated guideline for reporting systematic reviews. BMJ 2021;372:n71. doi: 10.1136/bmj.n71. For more information, visit: http://www.prisma‐statement.org/.

An electronic search strategy was performed by two independent reviewers (H.X.S.T. and K.Y.F.) on PubMed, EMBASE, Web of Science, and the Cochrane Controlled Register of Trials for relevant articles, with restriction to English language articles. The search was conducted from inception to July 31, 2023. The comprehensive search using the keywords “smartphone,” “cardiac implantable electronic device,” “remote monitoring,” and “outcomes,” “compliance,” “connectivity,” or “mortality.” The entire search strategy is listed in Table S1. Retrieved abstracts and full texts were reviewed by two independent investigators (H.X.S.T. and V.H.T.); conflicts were resolved via group consensus among all authors in the study. All studies comparing SM‐RM (intervention group) versus BM‐RM (control group) were included (Figure 1). Studies without BM‐RM (control group) arm, case reports, case series, review articles and conference abstracts were excluded.

Data from included studies were extracted by using a standardized data collection template with predefined data fields, including author, publication year, type of study and countries involved, number of participants in total and each study arm, age of participants, gender, device type, manufacturer, proportion of individuals on bedside monitoring, smart device operating system used, compliance, connectivity and episode transmission time. Where possible, efforts were made to contact the authors for more information.

## 3 RESULTS

The search strategy retrieved 3633 studies. After removing 411 duplicates, 3222 studies were screened according to title and abstract. Finally, two articles^17^, ^18^ were retrieved, which are summarized in Figure 1 and Tables 1 and 2.

**TABLE 1:** Baseline characteristics.

Author	Publication year	Date of study	Type of study; countries involved	Participants	Age: Mean (SD)	Female (%)	Follow up duration (days)
Manyam	2023	Patients who received implantation between Sept 2020 and June 2021, and followed up till July 2021	Retrospective, propensity‐score matched; USA	Total: 18794 SM‐RM: 9397 BM‐RM: 9397	SM‐RM: 69 (60‐77) BM‐RM: 69 (61‐77)	SM‐RM: 28 BM‐RM: 27	SM‐RM: 119 (75–180) BM‐RM: 126 (80–189)
Tarakji	2021	April to December 2018	Prospective (SM‐RM) and retrospective (BM‐RM); matched cohort; Multicenter international cohort (20 sites in the United States, the United Kingdom, France, and Italy)	Total: 3184 SM‐RM (pacemaker or CRT‐P): 245 BM‐RM (PM Manual: 979 PM wireless: 980 Defibrillator wireless: 980)	SM‐RM: 64.8 (15.6) BM‐RM (PM manual: 72.2 (11.5) PM wireless: 74.7 11.7 Defibrillator wireless: 66.4 (12.8))	SM‐RM: 41.6 BM‐RM (PM manual: 47.9 PM wireless: 46.2 Defibrillator wireless: 29.7)	Both SM‐RM and BM‐RM: 365 days

Abbreviations: BM, bedside monitoring; CRT, cardiac resynchronization therapy; CRT‐D, cardiac resynchronization therapy‐defibrillator; CRT‐P, cardiac resynchronization therapy‐pacemaker; ICD, implantable cardioverter‐defibrillator; PM, pacemaker; RM, remote monitoring; SD, standard deviation; SM, smart device monitoring.* before matched analysis.

**TABLE 2:** Results.

Author	Device type n (%)	Manufacturer	Patients on bedside monitoring (%)	Types of smart device and operating system used	Compliance	Connectivity	Episode transmission time
Manyam	CRT‐D: SM‐RM: 4267 (45) BM‐RM: 4328 (46) Dual chamber ICD: SM‐RM: 3452 (37) BM‐RM: 3552 (38) Single chamber ICD: SM‐RM: 1678 (18) BM‐RM: 1517 (16)	Abbott	50	Abbott‐provided smart phone or patients' own smartphone Android and iOS	SM‐RM: 98.1% BM‐RM: 94.3% *p* < .001	Frequency of data transmission: SM‐RM: Every 1.2 (1.1, 1.7) days; BM‐RM: Every 1.7 (1.5, 2.0) days; p< .001 Proportion of individuals who utilized patient‐initiated transmissions: SM‐RM: 55.6% BM‐RM: 28.1% *p* < .001 Median number of patient‐initiated transmissions sent per patient: SM‐RM: 1 (IQR: 1–3) BM‐RM: 1 (IQR: 1–2) *p* < .001 Transmitted ≥1: SM‐RM: 98.1% BM‐RM: 94.3% *p* < .01 Proportion of individuals who utilized patient‐initiated transmissions, by age: SM‐RM: Age < 75: 57.2% Age ≥ 75: 52.2% (*p* < .001) BM‐RM: Age < 75: 29.5% Age ≥ 75: 25.2% *p* < .001 Among population utilizing patient‐initiated transmissions, patients having ≥10 Transmissions: SM‐RM: 7% BM‐RM: 2% *p* < .001 Patients utilizing patient‐initiated transmissions in the first month SM‐RM: 96.7% BM‐RM: 98.5% Patients utilizing patient‐initiated transmissions at 6 months after implant SM‐RM: 4% BM‐RM: 3%	Episodes were viewed by a clinician within a similar amount of time [~1 (0.6–2) days] both in SM‐RM and BM‐RM
Tarakji	*SM‐RM: Single‐chamber: 9 (3.7) Dual‐chamber: 185 (75.5) CRT‐P/CRT‐D: 51 (20.8) *BM‐RM (PM manual: Single‐chamber: 10 550 (8.2) Dual‐chamber: 114 296 (88.9) CRT‐P/: 3761 (2.9) PM wireless: Single‐chamber: 4996 (7.2) Dual‐chamber: 55 911 (80.7) CRT‐P: 8406 (12.1) Defibrillator wireless: Single‐chamber: 10 052 (21.2) Dual‐chamber: 18 115 (38.2) CRT‐D: 19 290 (40.6)	Medtronic	92.3	Patients' own smart device (tablet or phone) iOS	Success rate of scheduled RM transmission: SM‐RM: 94.6% (91.8%–96.6%) (902/953) BM‐RM (PM manual transmission: 56.3% [53.7%–58.9%] [1531/2719] PM wireless: 77% [74.4%–79.4%] [2201/2859] Defibrillator wireless: 87.1% [85.2%–88.8%][3125/3587]		

Abbreviations: BM, bedside monitoring; CRT‐D, cardiac resynchronization therapy‐defibrillator; CRT‐P, cardiac resynchronization therapy‐pacemaker; ICD, implantable cardioverter‐defibrillator; IQR, interquartile range; PM, pacemaker; RM, remote monitoring; SM, smart device monitoring. * before matched analysis.

### 3.1 Study characteristics

Both studies were propensity score‐matched studies, published in 2021^18^ and 2023,^17^ respectively (Table 1).

Tarakji et al. used MyCareLinkHeartTM (Medtronic, Minneapolis, MN, USA) App as SM‐RM compared with BM‐RM in a primarily *pacemaker* (*PM*) *population*. The primary objective was to assess the success rate of scheduled RM transmissions among SM‐RM patients compared with patients in the historical control group. The control group consisted of manual communication using a wand with a bedside console (PM manual transmission). Moreover, post hoc analyses were performed to compare the success rate of scheduled RM transmissions between SM‐RM patients and patients in two additional historical control arms of automatic bedside consoles (PM patients with wireless automatic communication with the bedside console and defibrillator patients with similar automatic communication). A scheduled transmission was considered successfully completed if transmission was received by the manufacturer's central repository system within a window starting 1 day before and ending 5 days after the scheduled transmission date.

Manyam et al used the myMerlinPulse™ (Abbott, Chicago, IL, USA) App as SM‐RM, compared with the traditional BM‐RM (Merlin@Home™ transmitter) in a defibrillator population. The study objectives were as follows:

1. Compliance with RM was quantified as the proportion of patients enrolling in the RM system and transmitting data (scheduled or unscheduled) at least once.
2. Connectivity was measured by the median number of days between consecutive transmissions per patient.
3. Patient initiated transmission was measured by frequency of patient‐initiated transmission and median utilization of patient‐initiated transmission per patient.
4. Episode transmission time was measured as median time from episode detection to transmission and to availability within the manufacturer central repository system.

A total of 21 978 patients were being studied, of which 9642 (43.8%) were in the SM‐RM arm versus 12 336 (56.2%) in the BM‐RM arm. The mean age of patients ranged from 64 to 75 years, and females consisted of 27.0%–47.9%. Manyam et al. reported that the proportion of patients enrolled in RM was 94.4% (SM‐RM), compared to 85.0% (BM‐RM). Tarakji et al. reported that all SM‐RM patients enrolled in RM.

### 3.2 Study outcomes

#### 3.2.1 Compliance

Both studies found that SM‐RM led to more completed transmissions than BM‐RM. Manyam et al. found that more SM‐RM patients than BM‐RM patients transmitted at least once (98.1% vs. 94.3%, *p* < .001). Patients aged ≥75 had similar compliance rates for SM‐RM and BM‐RM as those aged <75. Tarakji et al. showed SM‐RM patients have higher success rates of scheduled transmissions than traditional BM‐RM methods, even after matching groups by age, type of device, and sex (SM‐RM: 94.6% (95% confidence interval, CI: 91.8%‐96.6%), PM manual: 56.3% (95% CI:53.7%‐ 58.9%), PM wireless: 77.0% (95% CI: 74.4% ‐ 79.4%), defibrillator wireless: 87.1% (95% CI: 85.2% ‐ 88.8%).

#### 3.2.2 Connectivity and patient‐initiated transmissions

Manyam et al. showed that SM‐RM (every 1.2 (1.1, 1.7) days) performed better than BM‐RM every 1.7 (1.5, 2.0) days) at connectivity (p<0.001; overall). More SM‐RM patients utilized patient‐initiated transmissions compared with BM‐RM (55.6% vs. 28.1%, *p* < .001). The median number of patient‐initiated transmissions sent per patient in the SM‐RM group was 1 (interquartile range [IQR], 1–3) and 1 (IQR, 1–2) in the BM‐RM group (*p* < .001). False‐positive transmissions were not mentioned by the article. More SM‐RM patients than BM‐RM patients transmitted at least once (98.1% vs. 94.3%, *p* < .001). Younger patients (aged <75) had more patient‐initiated transmissions compared with those aged ≥75 in both SM‐RM (57.2% vs. 52.2%, *p* < .001) and BM‐RM (29.5% vs. 25.2%, *p* < .001). There was a similar connectivity between men and women. Within the patient population utilizing patient‐initiated transmissions, 7% of the patients had 10 or more such transmissions in the SM‐RM group compared with 2% within the BM‐RM group (*p* < .001). Moreover, the percentage of patients utilizing patient‐initiated transmissions was significantly higher in the first month (SM‐RM: 96.7% and BM‐RM: 98.5%) compared with 6 months (SM‐RM: 4% and BM‐RM: 3%) after implant. Compliance within SM‐RM was further improved in patients utilizing the study's educational program. Additionally, within SM‐RM, patients using their mobile device had significantly better connectivity than those using manufacturer‐provided mobile devices.

#### 3.2.3 Episode transmission time

Manyam et al. highlighted that the median time from episode occurrence to the transmission of information to the application was significantly faster for SM‐RM than BM‐RM for clinical events. For auto mode switch/atrial tachyarrhythmia/atrial fibrillation, SM‐RM: median 4.2 days, IQR 1.10–13.7 days, BM‐RM: median 5.9 days, IQR 1.6–17.1 days, *p* < .001. For non‐sustained ventricular tachyarrhythmia/supraventricular tachycardia, SM‐RM: median 17.1 h, IQR 10.7–56.4 h, BM‐RM: median 24.3 h, IQR: 12.9–158.1 h, *p* < .001. For ventricular tachycardia or ventricular fibrillation, SM‐RM: median 13.1 h, IQR 7.31–23.0 h, BM‐RM: median 14.2 h, IQR 8.6–30.8 h, *p* = .005. The study also noted that episodes were viewed by a clinician within a similar amount of time both in SM‐RM and BM‐RM.

## 4 DISCUSSION

The important findings of this systematic review are as follows: (1) There is significantly higher compliance in patients with SM‐RM compared with BM‐RM in both pacemaker, and defibrillator patients; (2) SM‐RM is associated with higher connectivity; (3) more SM‐RM patients transmitted at least once (including patient‐initiated transmissions); (4) episodic transmission time of SM‐RM patients was shorter than that of BM‐RM patients. When looking at the comparison of median number of patient‐initiated transmissions sent per patient between SM‐RM and BM‐RM, there is similar median number among the two groups, with 1 (IQR 1–3) versus 1 (IQR 1–2), respectively. However, when looking at proportions, significantly more patients with SM‐RM are likely to transmit patient‐initiated transmissions compared to BM‐RM (55.6% vs. 28.1%, *p*<.001). This could be partly due to false‐positive transmissions, although this figure was not reported by the article.

Studies have shown that RM adherence rates, ranging from 49% to 89%, remained suboptimal despite mounting evidence showing that RM improves outcomes.^19^, ^20^, ^21^, ^22^ Those studies, however, were published before the COVID‐19 pandemic. During the recent COVID‐19 pandemic, telemedicine use has increased significantly, encouraging greater use of digital health technology in RM.^11^, ^12^, ^13^ The novel technology of using BLE (SM‐RM) potentially closing the gap by increasing convenience, higher RM compliance and connectivity as shown in this systematic review. SM‐RM is also popular as patients are increasingly tech‐savvy and carry smart devices with them.^6^ Being familiar with smart devices applications translates into easier use and a less steep learning curve for SM‐RM. Rather than requiring separate monitoring equipment at home for BM‐RM, smart device‐use is more convenient, making SM‐RM more accessible.

A key advantage of SM‐RM is the availability of a patient‐facing interface, allowing patients to play an active role in their treatment. Patients can analyze trends, access device data, or initiate transmissions anytime. Patients also report feeling better informed regarding their device, cardiac events, their overall health condition, and the process of RM.^23^ Being increasingly empowered and involved in their health may have contributed to increased compliance in the above studies. Moreover, SM‐RM enhances communication between patients and healthcare providers, motivating patient use. Additionally, the app provides access to educational material. Although some BM‐RM also have patient‐facing interfaces, they may not be as user‐friendly or convenient as smart devices applications.

From the healthcare provider's perspective, SM‐RM potentially further improves patient care and resource allocation by enabling quick response to events, reducing in‐person consultations and lowering costs and resources. Additionally, SM‐RM is expected to be more cost‐effective than BM‐RM, since it uses patients' existing smart devices rather than a bedside device. Utilizing patients' existing smart devices would reduce the marginal cost of purchasing an additional bedside device. There is a concern that SM‐RM may further increase healthcare providers' workload. This is so as more manpower might be needed to respond to patients' transmissions, and distinguish between true versus false‐positive patient‐initiated transmssions. However, the added burden seems to be limited: Manyam et al found that the percentage of patients utilizing patient‐initiated transmissions was significantly higher in both groups in the first month (SM‐RM: 96.7% and BM‐RM: 98.5%) when compared with 6 months (SM‐RM: 4% and BM‐RM: 3%) after implant. The reason provided by the authors being that the feedback from the device clinic, in response to the patient‐initiated transmissions that occurred during the first month, could have led to a lower rate of subsequent patient‐initiated transmissions. It could also be possible that patients' motivation towards treatment may have declined with time. Tarakji et al. showed that patients using the app sent fewer unscheduled transmissions (3 on average) than patients with traditional RM options, and nearly half of these patients did not send any (46.5%).

However, potential health disparities can be exacerbated by SM‐RM due to varied levels of smart device ownership and technological literacy. Lower socioeconomic status might be associated with poorer connectivity due to reduced smartphone or broadband usage.^17^ Increased financial burden from smart device or broadband purchase may deter individuals from adopting or complying with SM‐RM interventions. Geographical areas with poorer connectivity might also face challenges with SM‐RM adoption. Additionally, older patients might need help with SM‐RM and adapting to digital health technology.^1^, ^2^ Nonetheless, similar compliance and connectivity has been reported for older and younger individuals.^17^ This suggests that compliance with RM might not be restricted to age and familiarity with technology.^3^ A possible reason could be that younger individuals are busier at work than retirees and hence forget their scheduled transmissions.

To address these challenges, rather than completely transitioning to SM‐RM in the future, both BM‐RM and SM‐RM options can co‐exist. Additionally, comprehensive and personalized education and caregiver training can enhance the effectiveness of SM‐RM use,^1^ bridging healthcare disparities and reducing the workload on healthcare providers.

Another important issue is cybersecurity threat. Implanted CIED are at risk for direct and indirect interference with device function through malicious intrusion. Therefore, it is important that the central regulatory body (European Medicines Agency, EMA or Food and Drug Administration, FDA) plays an important role in evaluating CIEDs for potential cybersecurity issues through pre‐ and post‐market evaluation.^24^ To date, no cases of actual patient harm have ever been reported from the cybersecurity intrusion on CIED.

In the current literature, the clinical outcome and benefit of RM compared to in‐office monitoring remains unclear. Many RCTs^1^, ^3^, ^6^, ^25^, ^26^ and meta‐analyses^27^, ^28^ currently show conflicting results on mortality benefit and heart failure hospitalization between patients receiving RM and those with only in‐office follow up. With different studies showing varied outcomes, more research is necessary to better understand the clinical benefits of RM.

The main limitation of this study was the presence of only two relevant studies, and their confinement to only the United States and European countries. This may reduce validity and generalization to the entire world, given the marked discrepancies in healthcare technology usage globally. Discussion regarding the clinical benefits of SM‐RM was also limited as the source articles retrieved did not focus on this area in their research. Future research should focus on the clinical benefits of SM‐RM to understand its utility better. Meta‐analysis of the relationship between the effectiveness of SM‐RM in CIED patients compared to BM‐RM was not possible due to a low number of studies and different definitions used for outcome (compliance). Within our identified studies, there is heterogeneity in the outcomes reported, with different studies highlighting different strengths of SM‐RM in terms of types of smart devices used and operating system. Moreover, there are differences in the types of CIEDs in both articles, with Manyam et al. including CRT‐D and ICD, and Tarakji et al. including pacemakers, CRT‐D and defibrillators. In order to mitigate these limitations, future research comparing SM‐RM to BM‐RM with well‐defined and standardized outcomes is warranted.

## 5 CONCLUSION

In conclusion, SM‐RM appears to be beneficial. It improves compliance and connectivity, as well as allowing patients to feel empowered and in control of their health. Nevertheless, the choice between SM‐RM and BM‐RM should be guided by patients' individual preferences. Further research into the use of SM‐RM in a wider array of settings, and its cost‐effectiveness and long‐term clinical outcomes need to be carried out.

## FUNDING INFORMATION

No funding declared.

## CONFLICT OF INTEREST STATEMENT

Authors declare no conflict of interests for this article.

## Supporting information


Table S1.

## Data Availability

The data that support the findings of this study are available from the corresponding author upon reasonable request.
