# Supplementary material for: Remote monitoring of cardiac implantable electronic devices using smart device interface versus radiofrequency‐based interface: A systematic review
Source: J Arrhythm. 2024 May 9;40(3):596–604. doi: 10.1002/joa3.13054 (PMC11199811; doi:10.1002/joa3.13054)
Supplement: Supplementary file 1 — Table S1. [file JOA3-40-596-s001.docx]

**Remote Monitoring of Cardiac Implantable Electronic Devices Using Smart Device Interface versus Radiofrequency-based Interface: A Systematic Review**

**Supplementary Data**

Vern Hsen Tan^1^*, Hui Xin See Tow^2^*, Khi Yung Fong^2^, Wang Yue^1^, Colin Yeo^1^, Chi Keong Ching^3^, Toon Wei Lim^4^

^1^ Department of Cardiology, Changi General Hospital, Singapore

^2^ Yong Loo Lin School of Medicine, National University of Singapore, Singapore

^3^ Department of Cardiology, National Heart Centre Singapore, Singapore

^4^ Department of Cardiology, National University Heart Centre Singapore, Singapore

*Co-first author

Corresponding author:

Vern Hsen Tan, MBBS, MRCP, CCDS, CEPS

Senior Consultant, Department of Cardiology, Changi General Hospital, Singapore

Address: 2 Simei Street 3, Singapore 529889

Telephone: +65 6788 8833

Email: [tan.vern.hsen@singhealth.com.sg](mailto:tan.vern.hsen@singhealth.com.sg)

**Table S1: Search Strategy**

‌**Pubmed (81)**

| Smartphone OR mobile OR application OR app OR m-health  [1,903,902](https://pubmed.ncbi.nlm.nih.gov/?term=%28%28%28%28Smartphone%29+OR+%28mobile%29%29+OR+%28application%29%29+OR+%28app%29%29+OR+%28m-health%29&sort=) | #1 | "smartphone"[MeSH Terms] OR "smartphone"[All Fields] OR "smartphones"[All Fields] OR "smartphone s"[All Fields] OR "mobile"[All Fields] OR "mobiles"[All Fields] OR "applicabilities"[All Fields] OR "applicability"[All Fields] OR "application"[All Fields] OR "applications"[All Fields] OR "applicative"[All Fields] OR "australas plant pathol"[Journal] OR "app"[All Fields] OR "m-health"[All Fields] |
| --- | --- | --- |
| Remote monitor*  22,747 | #2 | ("remote"[All Fields] OR "remotely"[All Fields] OR "remoteness"[All Fields] OR "remotes"[All Fields]) AND "monitor*"[All Fields] |
| Cardiac Implantable Electronic Device OR pacemaker OR Implantable cardioverter defibrillator  83,520 | #3 | (("cardiacs"[All Fields] OR "heart"[MeSH Terms] OR "heart"[All Fields] OR "cardiac"[All Fields]) AND ("drug implants"[MeSH Terms] OR ("drug"[All Fields] AND "implants"[All Fields]) OR "drug implants"[All Fields] OR "implant"[All Fields] OR "embryo implantation"[MeSH Terms] OR ("embryo"[All Fields] AND "implantation"[All Fields]) OR "embryo implantation"[All Fields] OR "implantation"[All Fields] OR "implant s"[All Fields] OR "implantability"[All Fields] OR "implantable"[All Fields] OR "implantables"[All Fields] OR "implantate"[All Fields] OR "implantated"[All Fields] OR "implantates"[All Fields] OR "implantations"[All Fields] OR "implanted"[All Fields] OR "implanter"[All Fields] OR "implanters"[All Fields] OR "implanting"[All Fields] OR "implantion"[All Fields] OR "implantitis"[All Fields] OR "implants"[All Fields]) AND ("electronical"[All Fields] OR "electronically"[All Fields] OR "electronics"[MeSH Terms] OR "electronics"[All Fields] OR "electronic"[All Fields]) AND ("device s"[All Fields] OR "equipment and supplies"[MeSH Terms] OR ("equipment"[All Fields] AND "supplies"[All Fields]) OR "equipment and supplies"[All Fields] OR "device"[All Fields] OR "instrumentation"[MeSH Subheading] OR "instrumentation"[All Fields] OR "devices"[All Fields])) OR ("pacemaker s"[All Fields] OR "pacemaker, artificial"[MeSH Terms] OR ("pacemaker"[All Fields] AND "artificial"[All Fields]) OR "artificial pacemaker"[All Fields] OR "pacemaker"[All Fields] OR "pacemakers"[All Fields] OR "pacemaking"[All Fields]) OR ("defibrillators, implantable"[MeSH Terms] OR ("defibrillators"[All Fields] AND "implantable"[All Fields]) OR "implantable defibrillators"[All Fields] OR ("implantable"[All Fields] AND "cardioverter"[All Fields] AND "defibrillator"[All Fields]) OR "implantable cardioverter defibrillator"[All Fields]) |
| Compliance OR Connect* OR Outcome OR detection OR mortality OR hospitalization OR hospitalisation  11,655,931 | #4 | "compliances"[All Fields] OR "patient compliance"[MeSH Terms] OR ("patient"[All Fields] AND "compliance"[All Fields]) OR "patient compliance"[All Fields] OR "compliance"[All Fields] OR "compliance"[MeSH Terms] OR "connect*"[All Fields] OR ("outcome"[All Fields] OR "outcomes"[All Fields]) OR ("detect"[All Fields] OR "detectabilities"[All Fields] OR "detectability"[All Fields] OR "detectable"[All Fields] OR "detectables"[All Fields] OR "detectably"[All Fields] OR "detected"[All Fields] OR "detectible"[All Fields] OR "detecting"[All Fields] OR "detection"[All Fields] OR "detections"[All Fields] OR "detects"[All Fields]) OR ("mortality"[MeSH Terms] OR "mortality"[All Fields] OR "mortalities"[All Fields] OR "mortality"[MeSH Subheading]) OR ("hospital s"[All Fields] OR "hospitalisation"[All Fields] OR "hospitalization"[MeSH Terms] OR "hospitalization"[All Fields] OR "hospitalised"[All Fields] OR "hospitalising"[All Fields] OR "hospitality"[All Fields] OR "hospitalisations"[All Fields] OR "hospitalizations"[All Fields] OR "hospitalize"[All Fields] OR "hospitalized"[All Fields] OR "hospitalizing"[All Fields] OR "hospitals"[MeSH Terms] OR "hospitals"[All Fields] OR "hospital"[All Fields]) OR ("hospital s"[All Fields] OR "hospitalisation"[All Fields] OR "hospitalization"[MeSH Terms] OR "hospitalization"[All Fields] OR "hospitalised"[All Fields] OR "hospitalising"[All Fields] OR "hospitality"[All Fields] OR "hospitalisations"[All Fields] OR "hospitalizations"[All Fields] OR "hospitalize"[All Fields] OR "hospitalized"[All Fields] OR "hospitalizing"[All Fields] OR "hospitals"[MeSH Terms] OR "hospitals"[All Fields] OR "hospital"[All Fields]) |
| 81 | #1 AND #2 AND #3 AND #4 |  |
| **1576** |  | (Smartphone OR mobile OR application OR app OR m-health OR remote) AND (Cardiac Implantable Electronic Device OR pacemaker OR Implantable cardioverter defibrillator) AND (Compliance OR Connect* OR Outcome OR detection OR mortality OR hospitalization OR hospitalisation) NOT ((animals [mh] NOT humans [mh])) NOT (systematic[sb] OR Editorial[pt] OR Comment[pt] OR Meta-Analysis[pt] OR Letter[pt] OR Case Reports[pt] OR Clinical Conference[pt] OR Review[pt]) |

#1 AND #2 AND #3

102

(#1 OR #2) AND #3 AND #4

2362

**Embase (52)**

| Smartphone OR mobile OR application OR app OR m-health  1,538,884 | #1 | Smartphone OR mobile OR application OR app OR m-health |
| --- | --- | --- |
| Remote monitor*  26,785 | #2 | Remote monitor* |
| Cardiac Implantable Electronic Device OR pacemaker OR Implantable cardioverter defibrillator  57,882 | #3 | Cardiac Implantable Electronic Device OR pacemaker OR Implantable cardioverter defibrillator |
| Compliance OR Connect* OR Outcome OR detection OR mortality OR hospitalization OR hospitalisation  7,590,619 | #4 | Compliance OR Connect* OR Outcome OR detection OR mortality OR hospitalization OR hospitalisation |
| 52 | #1 AND #2 AND #3 AND #4 |  |
| **2018** |  | ('smartphone'/exp OR smartphone OR mobile OR 'application'/exp OR application OR 'app'/exp OR app OR 'm health' OR 'remote monitor') AND ('cardiac implantable electronic device'/exp OR 'pacemaker'/exp OR 'implantable cardioverter defibrillator'/exp) NOT ([conference abstract]/lim OR [conference paper]/lim OR [conference review]/lim OR [editorial]/lim OR [erratum]/lim OR [letter]/lim OR [review]/lim) |

#1 AND #2 AND #3

673

(#1 OR #2) AND #3 AND #4

22870

**Scopus (636)**

| Smartphone OR mobile OR application OR app OR m-health  28,308,713 | #1 | All Fields    Smartphone OR mobile OR application OR app OR m-health |
| --- | --- | --- |
| Remote monitor*  667,178 | #2 | ALL (remote AND monitor*) |
| Cardiac Implantable Electronic Device OR pacemaker OR Implantable cardioverter defibrillator  8,222 | #3 | ALL(cardiac AND implantable AND electronic AND device OR pacemaker OR implantable AND cardioverter AND defibrillator) |
| Compliance OR Connect* OR Outcome OR detection OR mortality OR hospitalization OR hospitalisation  24,443,029 | #4 | ALL (compliance OR connect* OR outcome OR detection OR mortality OR hospitalization OR hospitalisation ) |
| 636 | #1 AND #2 AND #3 AND #4 |  |
| **39** |  | TITLE-ABS-KEY ( ( smartphone OR mobile OR application OR app OR m-health OR 'remote AND monitor' ) AND ( cardiac AND implantable AND electronic AND device OR pacemaker OR implantable AND cardioverter AND defibrillator ) ) |
